# T2-mapping and T2*-mapping for detection of intramyocardial haemorrhage: a head-to-head comparison with T2-weighted imaging

**DOI:** 10.1186/1532-429X-17-S1-P80

**Published:** 2015-02-03

**Authors:** Pankaj Garg, Ananth Kidambi, David P Ripley, Adam K McDiarmid, Peter P Swoboda, Tarique A Musa, Bara Erhayiem, Laura E Dobson, John P Greenwood, Sven Plein

**Affiliations:** 1 Multidisciplinary Cardiovascular Research Centre & Leeds Institute for Cardiovascular and Metabolic Medicine, University of Leeds, Leeds, UK

## Background

A variety of CMR methods for detecting intramyocardial haemorrhage (IMH) has been proposed, including T2-weighted imaging (T2w), T2-mapping and T2* mapping. IMH detected by T2w imaging is associated with adverse LV remodelling and adverse outcome post acute myocardial infarction (MI). We compare the sensitivity, specificity, CNR and SNR of the three IMH imaging techniques.

## Methods

Twenty patients underwent CMR at 3T (Achieva TX system, Philips Healthcare, Best, The Netherlands) within 3 days following reperfused ST-elevation MI. Black blood, cine, T2w, T2-mapping, T2*-mapping and LGE imaging (0.1mmol/kg gadolinium DTPA) were performed in identical short axis locations using the ‘3 of 5' approach. Data were evaluated offline using commercial software (cvi42 v4.1.5, Circle Cardiovascular Imaging Inc., Calgary, Canada). On the LGE images showing the largest infarct volume, infarct size was determined by using a semi-automated histogram-based thresholding method. This slice was evaluated for visual presence of IMH by the three methods. Signal intensity (SI) and respective standard deviation of SI (SD) were measured for the infarcted myocardium, remote myocardium and any IMH (if present). SNR was computed for each using the formula=0.655((SI)/(SD)). CNR was determined comparing contrast-to-noise of infarcted myocardium to IMH (SNR_i_-SNR_IMH_).

## Results

**Figure 1:**
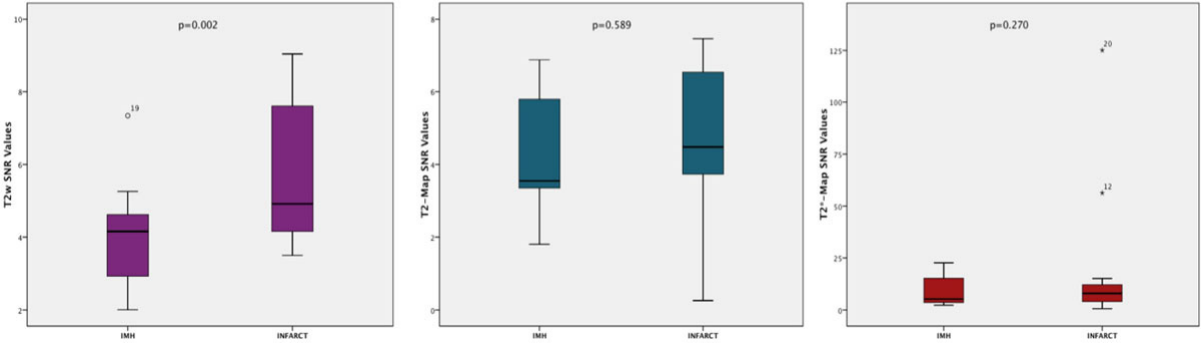
Box-plot of mean ± standard deviation (SD) of Signal-to-Noise-Ratio (SNR) for IMH and Infarct using the three imaging techniques.

Of the twenty patients, 55% (n=11) had IMH on T2w-imaging. The mean (±standard deviation) SNR and CNR values are listed in Table 1. The visual assessment of T2w imaging correlated strongly to T2-maps (r=0.69;p=0.001) and to the T2*-maps (r=0.60; p=0.005). The SNR for IMH and infarct zone were significantly different for only T2w imaging (Figure 1). Quantitative CNR for T2w imaging correlated strongly to visual assessment of all three imaging modalities (T2w- r=0.650; p=0.002, T2-map- r=0.454; p=0.04, T2*-map- r=0.603;p=0.005). The CNR for T2-maps and T2*-maps did not show similar correlation to the visual assessment.

**Table 1:** Mean ± standard deviation (SD) of SNR and CNR values for the three imaging modalities.

	T2-weighted	T2-maps	T2*-maps
SNR IMH	3.99±1.49	4.16±1.69	9.62±7.16

SNR Infarct	5.45±2.35	5.30±3.55	15.45±28.40

SNR Remote Myocardium	4.53±1.66	11.18±11.01	10.94±11.86

CNR	0.938±1.34	0.22±1.90	6.79±25.57

## Conclusions

Quantitative and qualitative T2w-imaging assessment for IMH is superior to T2-mapping and T2*mapping.

## Funding

JPG and SP receive a research grant from Philips Healthcare. SP is funded by British Heart Foundation fellowship (FS/10/62/28409).

